# Agreement and reliability of lower limb muscle architecture measurements using a portable ultrasound device

**DOI:** 10.3389/fphys.2022.981862

**Published:** 2022-09-02

**Authors:** Paul Ritsche, Reto Schmid, Martino V. Franchi, Oliver Faude

**Affiliations:** ^1^ Department of Sport, Exercise and Health, University of Basel, Basel, Switzerland; ^2^ Department of Biomedical Sciences, University of Padova, Padua, Italy; ^3^ CIR-MYO Myology Center, University of Padova, Padua, Italy

**Keywords:** ultrasound, muscle architecture, lumify, comparabality, reliability, lower limbs

## Abstract

High end ultrasonography devices lack in portability and are expensive. We investigated the agreement and reliability of a handheld and portable ultrasound system for human lower limb muscle architecture measurements. We captured ultrasound images of the rectus femoris (RF), vastus lateralis (VL) and gastrocnemius medialis (GM) in 36 active healthy participants (15 female, 21 male) at 50% of muscle length using the handheld Lumify (L12-4, linear-array 37 mm, Philips Healthcare, Amsterdam, Netherlands) and a high-end laboratory device (ACUSON Juniper, linear-array 54 mm, 12L3, SIEMENS Healthineers, Erlangen, Germany). We compared measurements of muscle fascicle length, pennation angle and thickness. To assess inter-session reliability of the Lumify system, participants were measured twice within 1 week. Comparing RF architecture measurements of both devices resulted in intra-class correlations (ICCs) ranging from 0.46–0.82 and standardized mean difference (SMDs) ranging from −0.45–0.05. For VL, ICCs ranged from 0.60–0.89 and SMDs ranged from −0.11–0.13. ICCs and SMDs for the GM ranged from 0.82–0.86 and −0.07–0.07. Calculating inter-session reliability for RF resulted in ICCs ranging from 0.44–0.76 and SMDs ranging from −0.38–0.15. For VL, ICCs and SMDs ranged from 0.57–0.75 and −0.13–0.02. ICCs for GM ranged from 0.75–0.92 and SMDs ranged from −0.15–0.16. Measurement of muscle thickness demonstrated the highest agreement (ICC ≥0.82) and reliability (ICC ≥0.75) across all muscles. The Lumify system was comparable to a high-end device and reliable for GM measurements. However, agreement and reliability were lower for the RF and VL. Of all evaluated architectural parameters, muscle thickness exhibited highest agreement and reliability.

## 1 Introduction

Modern day, high-end ultrasonography devices still are relatively expensive and bound to elevators and flat underground for transportation. Although most devices can be moved between assessment rooms, fast transportation across longer distances as well as availability for immediate assessment in the field is limited. Recently, ultrasonography devices consisting solely of a probe, an app, and/or a mobile display were introduced. Exemplars of these devices are, among others, the Philipps Lumify (Philips Healthcare, Amsterdam, Netherlands), the GE Healthcare Vscan Air (GE Medical Systems, Chicago, United States) or the Sonosite Iviz (FUJIFILM Sonosite, Bothell, United States). The devices usually weigh under 500 g, and thus inherit (nearly) unrestrained portability (Toscano et al., 2020). Furthermore, they are cheaper, with purchase prices usually being less than a quarter of high-end devices.

Overall, ultrasonography has many potential applications, and several investigations already examined the feasibility of using portable probes. Toscano et al. (2020) reported high sensitivity and specificity when investigating basic gynecology pathologies using the Lumify or Iviz probes. Johnson et al. (2022) on the other hand determined the Lumify probe to be accurate for measurements of the optic nerve sheath diameter in simulation models. Moreover, other investigations demonstrated that the Lumify probe can be used for point-of-care ultrasound training (Drake et al., 2021) and plastic surgery (Miller et al., 2018).

Apart from these applications, ultrasonography can be used to assess muscle architecture (Esformes et al., 2002; Narici et al., 2003; Franchi et al., 2018; Sarto et al., 2021). Ultrasonography measurements of muscle architecture are valid and reliable (Blazevich et al., 2006; Bénard et al., 2009; Bolsterlee et al., 2015; Scott et al., 2017; Geremia et al., 2019; Franchi et al., 2020; Nijholt et al., 2020). The architecture of a muscle is defined as arrangement of a muscle’s fibers relative to the force generation axis and characterized by fascicle length, pennation angle and thickness (Gans and Bock, 1965; Lieber and Frieden, 2000). Muscle architecture, especially of the lower limb, is not only important to aspects of physical performance (Sarto et al., 2021), but also to clinical outcomes (Onambele et al., 2006; Onambélé et al., 2007; Puthucheary et al., 2013; Stenroth et al., 2015; Paillard, 2017; van Alfen et al., 2018; Narici et al., 2021).

Yet, to the best of our knowledge, the comparability and reliability of portable probes to assess muscle architectural parameters has not been investigated. This however is relevant for both, a sports performance and medical context. Portable probes would allow for cost-effective, on-site athlete or patient screenings of muscle architecture as well as diagnosis of pathologies or injury. Furthermore, portable probes can be easily transported to allow for uncomplicated assessment of athletes belonging to different teams or patients in different hospital units. Moreover, due to lower acquisition cost, portable probes might facilitate a broader usage of ultrasonography to assess muscle architecture. This could lead to a better understanding of the significance of muscle architecture and its relation to performance and clinical outcomes. Therefore, the aim of this investigation was to determine the comparability and reliability of a portable probe (Philips Lumify) for lower limb muscle architecture measurements. For this, we compared the portable probe measurements to those of a high-end device and assessed test-retest reliability of the portable probe.

## 2 Materials and Methods

We conducted B-mode ultrasonography measurements of lower limb muscle architecture in 36 participants (15 female (age: 25.9 ± 2.7 years, height: 166.7 ± 3.9 cm, body mass: 59.9 ± 5.8 kg, skeletal muscle mass: 25.7 ± 2.8 kg, fat mass: 13.3 ± 2.9 kg), 21 male (age: 31.5 ± 7.0 years, height: 177.7 ± 7.2 cm, body mass: 71.5 ± 8.6 kg, skeletal muscle mass: 34.5 ± 5.0 kg, fat mass: 10.6 ± 4.5 kg)). Participants were required to be older then 18 years, healthy and without injury of the lower limbs within the prior 6 months. We asked the participants to refrain from exercise 24 h prior to the measurements. The study protocol was approved by the local ethics committee (Ethics Committee of North-Western and Central Switzerland, approval number: 2020–02,034) and complied with the Declaration of Helsinki. Participants signed an informed written consent prior to the start of the study after receiving all relevant study information.

To test the agreement of a 37-mm linear array portable probe (L12-4, Philips Healthcare, Amsterdam, Netherlands), we used one high-end ultrasonography device with a 56-mm linear array probe (12L-3, Acuson Juniper, SIEMENS Healthineers, Erlagen, Germany) as the gold standard. We used a Samsung Galaxy Tab S6 (Samsung, Seoul, South Korea) as mobile display in combination with the portable Lumify probe.

**FIGURE 1:**
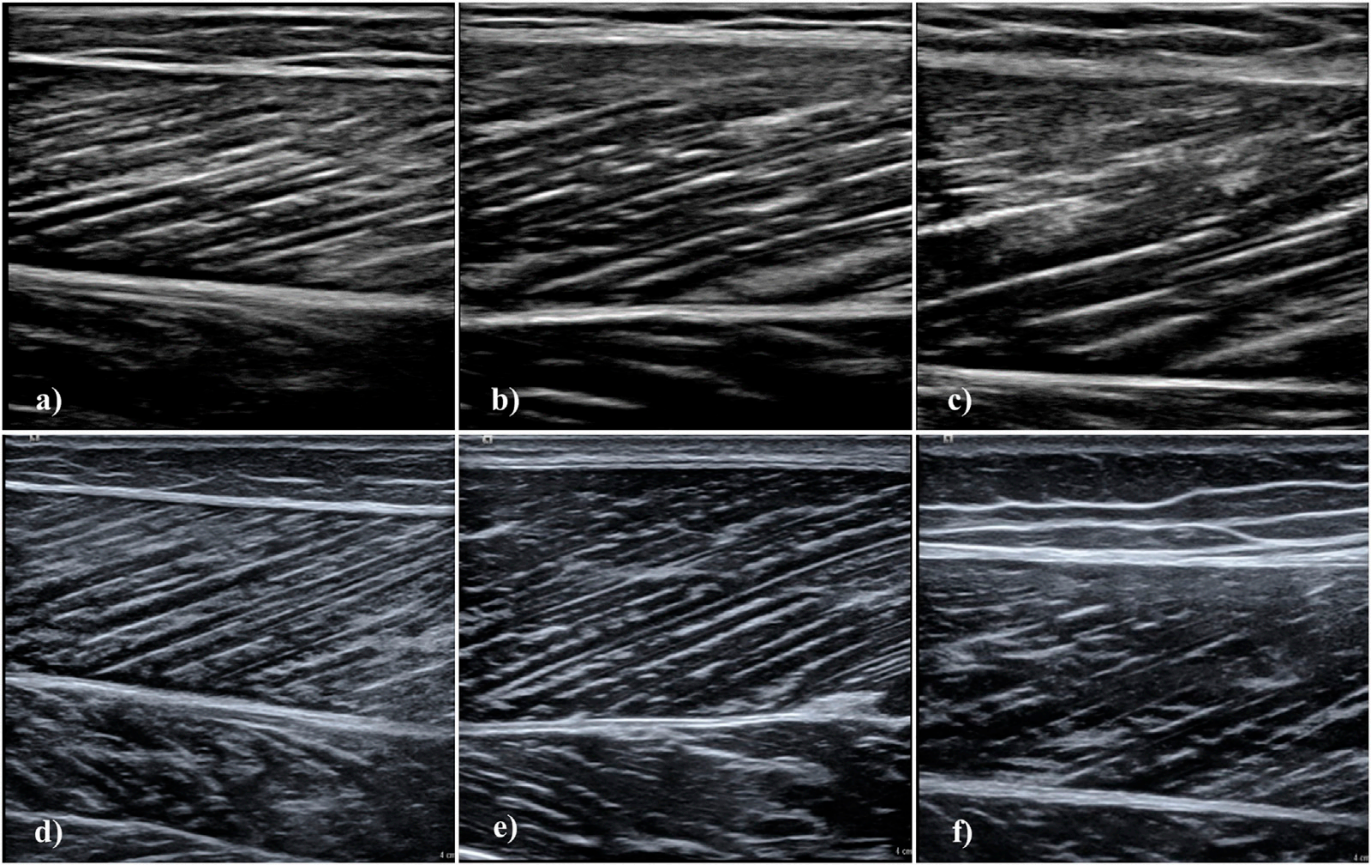
Longitudinal muscle ultrasonography images using the Philips Lumify and Siemens Acuson Juniper devices. Images acquired with the Lumify are **(A)** m. gastrocnemius medialis, **(B)** m. vastus lateralis, and **(C)** m. rectus femoris. Images acquired with the Acuson Juniper are **(D)** m. gastrocnemius medialis, **(E)** m. vastus lateralis, and **(F)** m. rectus femoris. Images displayed here were acquired in different participants, except for m. gastrocnemius medialis images.

Subsequently to collection of anthropometric data, we acquired longitudinal, static ultrasonography images of the m. rectus femoris, m. vastus lateralis and m. gastrocnemius medialis. We selected these muscles as they are the most relevant (and most investigated) to functional and clinical outcomes (Onambele et al., 2006; Onambélé et al., 2007; Puthucheary et al., 2013; Stenroth et al., 2015; Paillard, 2017; van Alfen et al., 2018; Narici et al., 2021; Sarto et al., 2021). Participants rested in a supine position for 5 minutes prior to acquisition of rectus femoris and vastus lateralis images. First, we determined and marked 50% of the distance between the proximal and distal muscle tendon junction. Moreover, we marked the muscle midpoint at this location as the middle of the distance between the medial and lateral muscle border assessed by ultrasonography. Following the placement on the region, we adapted the orientation of the probe according to fascicle plane (Bolsterlee et al., 2016). Subsequently, we took three images per muscle at this location (Figure 1) (Reeves et al., 2009; Franchi et al., 2015).

For gastrocnemius medialis measurement, participants were transitioned to a dynamometer (Isomed 2000; D. & R. Ferstl GmbH, Hemau, Germany) and placed in a prone position and rested for 5 minutes. The right foot was secured at an angle of 25° plantar flexion and we determined 50% of the distance between the proximal and distal muscle tendon junction (Narici et al., 2003). Again, we assessed and marked medio-lateral muscle midpoint, orientated the probe according to muscle fascicle orientation and acquired three images (Figure 1) (Narici et al., 2003; Bolsterlee et al., 2016). We employed a cross-over design for all muscles, always acquiring images with the high-end device first. We used the same marked locations upon collection of the images with the Lumify probe.

Furthermore, we assessed the test-retest reliability of the Lumify probe. Therefore, participants were invited for a second investigation 1 week later and three images per muscle were collected with the portable probe. Participants reported to the laboratory at the same time of day (± 1 hour). Re-marking of the muscle tendon junctions and muscle midpoints was necessary, as we did not instruct the participants to remark the locations by themselves.

All images were collected by an experienced investigator (PR) with 4 years of experience in acquiring and analyzing ultrasonography images (more than 1,000 acquired images of each of the here investigated muscles). We used a semi-automated tool SMA software (Seynnes and Cronin, 2020) to analyze the images and assess parameters muscle thickness, fascicle length and pennation angle. For each parameter, we computed the mean of at least two images. For every image, SMA computes the dominant fascicle orientation (Seynnes and Cronin, 2020). Then, fascicle length and pennation angle are calculated using the computed orientation and the two detected aponeuroses (Seynnes and Cronin, 2020). Muscle thickness is calculated as the mean distance between the two detected aponeuroses (Seynnes and Cronin, 2020). We used the maximum orientation value in each parameter and one value is calculated for each parameter for further analysis (Seynnes and Cronin, 2020). Analysis results were visually inspected and the analysis parameters were adapted in case the result was erroneous. It was demonstrated SMA is comparable to manual analysis (current gold standard for muscle architecture analysis) in the assessment of muscle thickness, fascicle length and pennation angle (Seynnes and Cronin, 2020).

### 2.1 Statistics

All statistical analyses were performed using R software (R Core Team, 2020) (rstudioapi, BlandAltmanLeh, readxl, irr, MBESS packages). Normality was assessed for all parameters using visual inspection (scatterplots, histograms and QQ-plots) and Shapiro-Wilk tests. We compared the portable probe measurement results to the high-end device measurement results for all muscles. For this purpose, we calculated consecutive-pairwise intra-class correlations (ICC), standard errors of measurement (SEM) and percentage standard errors of measurement (SEM%) with 95% compatibility intervals (CI). We classified the ICC values according to Koo and Li (2016) with ICC values of less than 0.5, between 0.5 and 0.75, between 0.75 and 0.9, and greater than 0.9 are poor, moderate, good and excellent (Koo and Li, 2016). We used Bland–Altman analysis (Bland and Altman, 1986) to test the measurement agreement of the portable probe with the high-end device. We set the limits of agreement to ± 1.96 standard deviations (SD). We calculated the standardized mean bias according to Hopkins et al. (2009), with 0.1, 0.3, 0.6, 1.0 and 2.0 being small, moderate, large, very large and extremely large errors. Furthermore, we investigated test-retest reliability of the portable probe by calculating ICCs, SEMs, SEM% with 95% CI. We classified the ICC values according to Koo and Li (2016) with ICC values of less than 0.5, between 0.5 and 0.75, between 0.75 and 0.9, and greater than 0.9 indicating poor, moderate, good and excellent reliability (Koo and Li, 2016). We calculated mean bias and standardized mean bias between test sessions. We categorized standardized mean bias as 0.1, 0.3, 0.6, 1.0 and 2.0 for extremely high, very high, high, moderate, and low reliability (Hopkins et al., 2009).

## 3 Results

**FIGURE 2:**
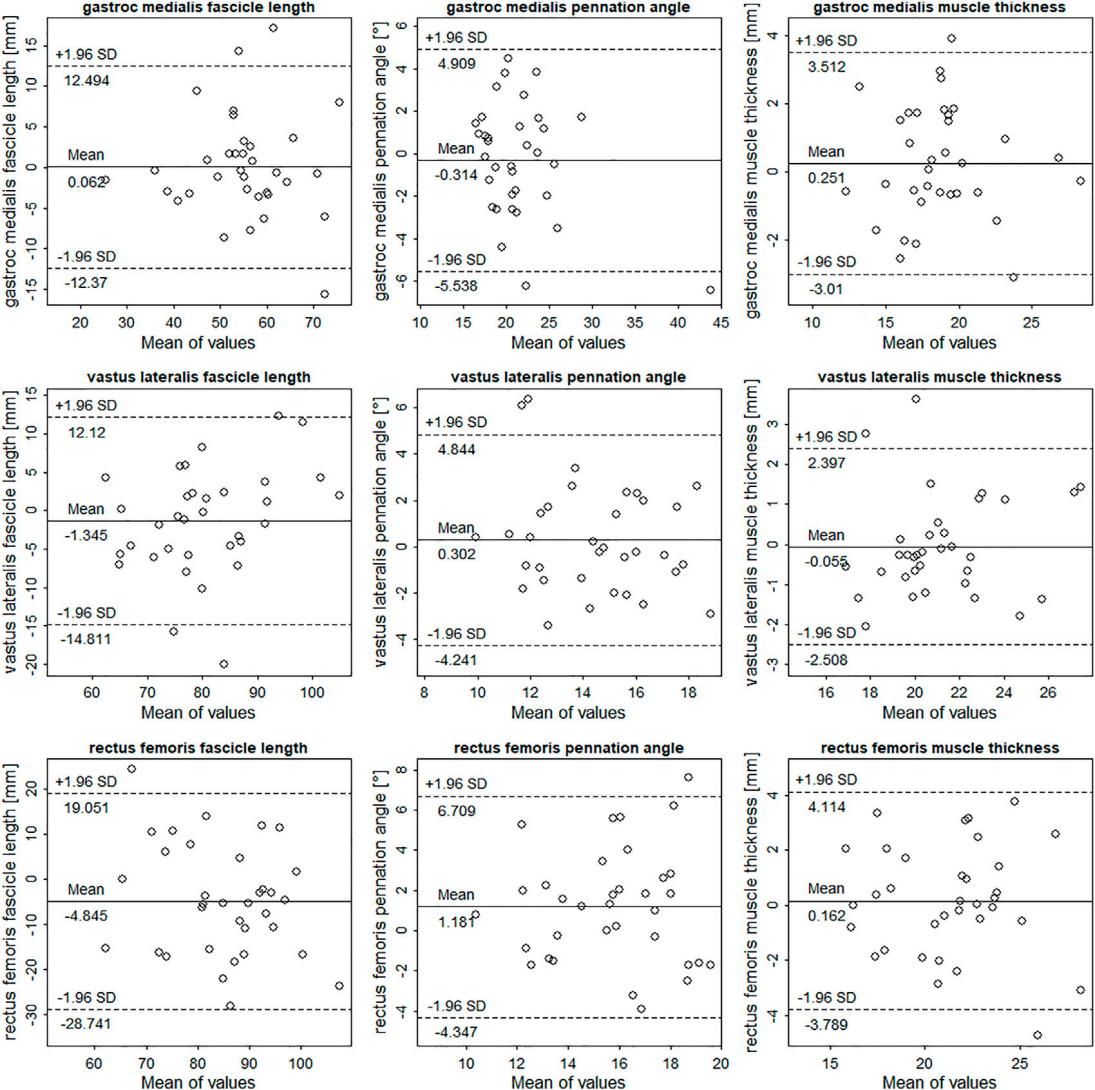
Bland-Altman plots comparing the Philips Lumify measurements to measurements of the Siemens Acuson Juniper (here taken as gold-standard). The differences between measurements are plotted against measurement means. Dotted and solid lines illustrate 95% limits of agreement and bias.

Mean values, ICCs, SEMs, SEM%, mean bias, and standardized mean bias of all muscles comparing the portable probe to the high-end device are shown in Table 1. Overall, the gastrocnemius medialis displayed the highest agreement between the two device types and rectus femoris the lowest. Measurement errors of the portable probe for gastrocnemius medialis were small in all parameters. In contrast, measurement errors of the portable probe for vastus lateralis were small for muscle thickness, whereas errors for fascicle length and pennation angle were moderate. Measurement of the portable probe errors for rectus femoris were large for fascicle length and pennation angle, yet muscle thickness measurement errors were small. Considering ICC values, portable probe assessment of architectural parameters in the gastrocnemius medialis according to Koo and Li (2016) ranged from moderate to excellent (95% CIs included). For the rectus femoris and vastus lateralis, ICC values can be classified as low to excellent (95% CIs included), demonstrating a large variability between parameters. Taking the investigated muscles together, ICCs, SEM%, and standardized mean bias for fascicle length ranged from 0.457 to 0.899, 6–10.7%, and –0.45 to 0.01. For pennation angle, ICCs, SEM%, and standardized mean bias ranged from 0.423 to 0.865, 8.4–12.6%, and −0.07 to 0.43. Comparison of muscle thickness resulted in ICCs, SEM%, and standardized mean bias ranges of 0.821–0.889, 4.4–6.6%, and −0.02 to 0.07. Results of the Bland-Altman analysis can be found in Figure 2. There is no indication of heteroscedasticity in all muscles and parameters. Rectus femoris fascicle length seems to be slightly underestimated by the Lumify probe, whereas rectus femoris pennation angle seems to be slightly overestimated (Figure 2).

**TABLE 1:** Comparability statistics of the portable Lumify system and the high-end device.

Parameter	Mean value Lumify	Mean value high-end	ICC	SEM	SEM%	Mean Bias	SMD
GM FL	55.0 ± 11.2	55.1 ± 11.0	0.84 (0.71,0.92)	4.5 (3.0,5.7)	7.8	0.1 (−12.4,12.5)	0.1 (−0.4,0.5)
GM PA	21.7 ± 5.5	21.4 ± 4.6	0.87 (0.75,0.93)	1.9 (1.4,2.3)	8.4	−0.3 (-5.5,4.9)	−0.1 (−0.5,0.4)
GM MT	18.6 ± 3.5	18.9 ± 3.4	0.89 (0.79,0.94)	1.2 (0.9,1.4)	6.6	0.3 (-3.0,3.5)	0.1 (-0.4,0.6)
RF FL	87.5 ± 13.5	82.7 ± 10.9	0.46 (0.15,0.69)	8.6 (6.7,10.3)	10.7	−4.8 (−28.7,19.1)	−0.5 (−0.9,0.1)
RF PA	15.2 ± 2.7	16.4 ± 2.8	0.42 (0.11,0.66)	1.9 (1.5,2.4)	12.5	1.2 (−4.4,6.7)	0.4 (−0.1,0.9)
RF MT	21.2 ± 3.4	21.4 ± 3.2	0.82 (0.67,0.91)	1.4 (1.1,1.7)	6.5	0.2 (-3.8,4.1)	0.1 (-0.4,0.5)
VL FL	81.2 ± 9.9	79.9 ± 12.0	0.80 (0.64,0.90)	4.9 (3.4,6.1)	6	−1.4 (−14.8,12.1)	−0.1 (−0.6,0.4)
VL PA	14.3 ± 2.8	14.6 ± 2.4	0.60 (0.33,0.78)	1.6 (1.2,2.0)	12.6	0.3 (−4.2,4.8)	0.1 (-0.4,0.6)
VL MT	21.2 ± 2.5	21.1 ± 2.7	0.89 (0.79,0.94)	0.9 (0.6,1.1)	4.3	−0.1 (−2.5,2.4)	−0.1 (−0.5,0.5)

All values calculated for m. gastrocnemius medialis (GM), m. rectus femoris (RF) and m. vastus lateralis (VL) comparing the Lumify probe to a high-end device. Parameters of comparison were fascicle length in mm (FL), pennation angle in ° (PA) and muscle thickness in mm (MT). Mean values ± standard deviation. Consecutive pairwise intra-class correlation coefficient (ICC) and standard error of measurement (SEM) with 95% compatibility interval. Standard error of measurement is also displayed in percent (SEM%). Mean bias and standardized mean bias (SMD) with limits of agreement set to ± 1.96 standard deviations.

Mean values, test-retest ICCs, SEMS, SEM%, mean bias, and standardized mean bias of all muscles for the portable probe are shown in Table 2. According to Hopkins et al. (2009), reliability of the portable probe to assess gastrocnemius medialis muscle thickness, fascicle length, and pennation angle, was extremely high to very high. For vastus lateralis architectural parameters, reliability of the portable probe was extremely high to very high. The portable probe demonstrated very high to high reliability for rectus femoris architecture assessment. Nonetheless, considering ICC values, reliability of the portable probe to assess architectural parameters in the gastrocnemius medialis according to Koo and Li (2016) ranged from moderate to excellent (95% CIs included). For the rectus femoris and vastus lateralis, reliability ranged from low to excellent (95% CIs included), demonstrating a large variability between parameters. Taking the investigated muscles together, ICCs, SEM%, and standardized mean bias for fascicle length ranged from 0.439 to 0.789, 7–11.8%, and −0.15 to 0.15. For pennation angle, ICCs, SEM%, and standardized mean bias ranged from 0.520 to 0.746, 9.4–12.7%, and -0.38 to 0.16. Comparison of muscle thickness resulted in ICCs, SEM%, and standardized mean bias ranges of 0.746–0.921, 5.7–7.8%, and −0.19 to −0.02.

**TABLE 2:** Test-retest statistics for the Lumify probe.

Parameter	Mean MTP 1	Mean MTP 2	ICC	SEM	SEM%	Mean Bias	SMD
GM FL	55.0 ± 11.2	55.6 ± 8.8	0.79 (0.62,0.89)	4.6 (3.2,5.7)	8.5	−1.6 (−3.9,0.6)	−0.2 (−0.6,0.3)
GM PA	21.7 ± 5.5	20.8 ± 4.4	0.75 (0.55,0.86)	2.5 (1.4,3.6)	9.3	0.9 (−0.3,2.1)	0.2 (-0.3,0.6)
GM MT	18.6 ± 3.5	18.6 ± 3.1	0.92 (0.85,0.96)	0.9 (0.7,1.2)	5.7	−0.1 (−0.5,0.4)	−0.1 (−0.5,0.5)
RF FL	87.5 ± 13.5	85.6 ± 15.1	0.44 (0.13,0.67)	10.8 (7.6,13.3)	11.8	1.9 (−3.4,7.3)	0.2 (−0.3,0.6)
RF PA	15.2 ± 2.7	16.2 ± 3.1	0.52 (0.23,0.73)	1.9 (1.5,2.3)	11.9	−1.0 (−2.0,−0.1)	−0.4 (−0.9,0.1)
RF MT	21.2 ± 3.4	21.9 ± 3.4	0.76 (0.57,0.87)	1.6 (1.2,2.1)	7.8	−0.6 (−1.5,0.2)	−0.2 (−0.7,0.3)
VL FL	81.2 ± 9.9	81.1 ± 11.4	0.72 (0.51,0.85)	5.7 (4.2,7.0)	7.0	0.2 (−2.7,3.0)	0.1 (−0.5,0.5)
VL PA	14.3 ± 2.8	14.7 ± 2.4	0.57 (0.3,0.76)	1.7 (1.3,2.0)	12.7	−0.4 (−1.2,0.5)	−0.1 (−0.6,0.4)
VL MT	21.2 ± 2.5	21.3 ± 2.9	0.75 (0.55,0.86)	1.4 (1.0,1.7)	6.9	−0.1 (−0.8,0.6)	−0.1 (−0.5,0.4)

All values calculated for m. gastrocnemius medialis (GM), m. rectus femoris (RF) and m. vastus lateralis (VL). Parameters of comparison were fascicle length in mm (FL), pennation angle in ° (PA) and muscle thickness in mm (MT). Measurement timepoint (MTP) mean values ± standard deviation. Consecutive pairwise intra-class correlation coefficient (ICC) and standard error of measurement (SEM) and mean bias with 95% compatibility interval. Standard error of measurement is also displayed in percent (SEM%). Standardized mean bias (SMD) with limits of agreement set to ± 1.96 standard deviations.

## 4 Discussion

Our results indicate that the portable Lumify probe demonstrated small to moderate measurement errors compared to the high-end device with low to excellent ICC values. Test-retest reliability considering standardized mean bias was extremely high to high and ICC values were low to excellent. Whereas agreement and reliability were highest for the measurement of muscle thickness across all muscles, pennation angle measurement in gastrocnemius medialis and vastus lateralis and fascicle length measurement in rectus femoris presented the lowest agreement and reliability. Muscle specific agreement and reliability were highest for gastrocnemius medialis.

Although several studies investigated the test-rested reliability and validity of ultrasonographic measurement of muscle architecture (Blazevich et al., 2006; Bénard et al., 2009; Bolsterlee et al., 2015; Scott et al., 2017; Geremia et al., 2019; Franchi et al., 2020; Nijholt et al., 2020), few tested the agreement of different probes (Cho et al., 2018; Nijholt et al., 2020). Nijholt et al. (2020) compared the measurements of a linear and curved array probe and tested the validity of lower limb muscle thickness, cross-sectional area and echo intensity against MRI. They reported both, the linear and curved array probe, to be reliable (ICC between 0.87 and 0.97) and valid (mean difference between 0.2 and 2.1 cm^2^). Cho et al. (2018) investigated the inter- and intra-rater reliability of a dual-probe ultrasonography system and compared the results to a standard ultrasonography device. They reported excellent reliability and agreement for gastrocnemius medialis pennation angle (ICCs >0.9 and SEMs <1°). In contrast to our results, the agreement and validation values reported in these studies indicate higher agreement between the investigated systems. Moreover, reported agreement in validation studies of ultrasonography based muscle architecture assessment using cadaveric dissection was higher as well (Bénard et al., 2009; Kellis et al., 2009). In terms of test-retest reliability of ultrasonographic architecture assessment in all here investigated muscles, ICCs between 0.7 and 0.98, 0.7 to 0.98 and 0.74 to 0.99 have been reported for muscle thickness, fascicle length, and pennation angle, respectively (Narici et al., 2003; Blazevich et al., 2006; Ema et al., 2013; Bolsterlee et al., 2015; Trezise et al., 2016; Geremia et al., 2019; Nijholt et al., 2020; Betz et al., 2021; May et al., 2021; Hagoort et al., 2022). Reported SEM% were between 0.6 and 4.8%, 2–18.9%, and 4.3–23% for muscle thickness, fascicle length, and pennation angle, respectively (Narici et al., 2003; Bénard et al., 2009; Ema et al., 2013; Bolsterlee et al., 2015; Trezise et al., 2016; Oranchuk et al., 2020; May et al., 2021; Hagoort et al., 2022). Compared to the existing literature, solely our gastrocnemius medialis test-rest reliability ICC values for are within the ranges demonstrated in the literature (0.75–0.92 vs. 0.7–0.99). Our test-retest reliability ICC values for rectus femoris and vastus lateralis architecture were lower compared to reported values in the literature (0.44–0.76 vs. 0.7–0.99). Except for muscle thickness measurements, all our test-retest reliability SEM% values are within the ranges demonstrated in the literature. Lower test-retest reliability of the Lumify probe might be explained by inferior image quality compared to high-end devices. Usually, reduced image quality inherits lower pixel contrast and increased signal noise, leading to less visible tissue structures. Because the SMA tool employs spatial filters to detect muscle architectural parameters and aponeuroses, lower image quality thus leads to less accurate detection and therefore less accurate image evaluation.

Apart from the cost-effectiveness and portability advantages of portable probes, there are also some restraints. The extended-field-of-view modality (Weng et al., 1997) is usually not embedded in portable probes. In combination with the generally reduced transducer width, this limits the applicability of portable probes as fascicle length extrapolation is generally necessary which might result in measurement errors (Franchi et al., 2020). Moreover, assessment of muscle anatomical cross-sectional area in larger muscles (such as vastus lateralis, gastrocnemius medialis or rectus femoris) is therefore impossible using portable probes, except when single images are stitched together. In addition, the processing capabilities of portable probes are limited compared to high end devices, leading to less sophisticated signal post processing. Moreover, less parameters to adapt during image acquisition are available, limiting the options to ideally configure the imaging settings.

The here presented investigation has several limitations. First, we did not use cadaveric dissection as a gold standard to validate the portable probe measurements against, but a high-end device. However, using cadaveric dissection is expensive and access limited. Moreover it was previously demonstrated that ultrasonography is comparable to muscle architectural measurements in cadaveric samples (Bénard et al., 2009; Kellis et al., 2009). We did not use the extended-field-of-view ultrasonography modality to acquire longitudinal muscle images. Even though this might have led to increased accuracy (Franchi et al., 2018; Franchi et al., 2020), this modality is not embedded in the Lumify probe and the analysis software used is not specificized on analyzing extended-field-of-view images (Seynnes and Cronin, 2020). Because we used a fixed scanning order (high-end device first), longer resting period prior to measurements with the Lumify device might have resulted in fluid shifts that could have influenced our results. Nonetheless, no consistent bias is visible in our data. Furthermore, because we did not instruct participants to remark the scanning location of the first session, re-marking could have resulted in slightly altered scanning locations. This might have influenced the test-retest reliability of the Lumify device. Lastly, we only included healthy active adults in our investigation. This might limit the generalizability of our results to other populations.

## 5 Conclusion

To sum up, the portable Lumify probe demonstrated reliable muscle architecture measurements of lower limb muscles that are comparable to those of a high-end device. Highest reliability and agreement were observed for m. gastrocnemius measurements, lowest for m. rectus femoris. Nonetheless, measurement errors should be considered when interpreting observed longitudinal changes in muscle architecture assessed with the Lumify system. Future investigations should consider including different participant populations, comparing the reliability of different portable systems for muscle architecture assessment as well as including different muscle groups.

## Data Availability

The raw data supporting the conclusions of this article will be made available by the authors upon request, without undue reservation.

## References

[B1] Bénard M. R. Becher J. G. Harlaar J. Huijing P. A. Jaspers R. T. (2009). Anatomical information is needed in ultrasound imaging of muscle to avoid potentially substantial errors in measurement of muscle geometry. Muscle Nerve 39, 652–665. 10.1002/mus.21287 19291798

[B2] Betz T. Wehrstein M. Preisner F. Bendszus M. Friedmann-Bette B. (2021). Reliability and validity of a standardised ultrasound examination protocol to quantify vastus lateralis muscle. J. Rehabil. Med. 53, jrm00212. 10.2340/16501977-2854 34121129 PMC8638746

[B3] Bland M. J. Altman D. G. (1986). Statistical methods for assessing agreement between two methods of clinical measurement. Lancet 1, 307–310. 10.1016/s0140-6736(86)90837-8 2868172

[B4] Blazevich A. J. Gill N. D. Zhou S. (2006). Intra- and intermuscular variation in human quadriceps femoris architecture assessed *in vivo* . J. Anat. 209, 289–310. 10.1111/j.1469-7580.2006.00619.x 16928199 PMC2100333

[B5] Bolsterlee B. Gandevia S. C. Herbert R. D. (2016). Ultrasound imaging of the human medial gastrocnemius muscle: How to orient the transducer so that muscle fascicles lie in the image plane. J. Biomech. 49, 1002–1008. 10.1016/j.jbiomech.2016.02.014 26905734

[B6] Bolsterlee B. Veeger H. E. J. DirkJan) van der Helm F. C. T. Gandevia S. C. Herbert R. D. (2015). Comparison of measurements of medial gastrocnemius architectural parameters from ultrasound and diffusion tensor images. J. Biomech. 48, 1133–1140. 10.1016/j.jbiomech.2015.01.012 25682540

[B7] Cho J.-E. Cho K. H. Yoo J. S. Lee S. J. Lee W.-H. (2018). Reliability and validity of a dual-probe personal computer-based muscle viewer for measuring the pennation angle of the medial gastrocnemius muscle in patients who have had a stroke. Top. Stroke Rehabil. 25, 6–12. 10.1080/10749357.2017.1383723 28980515

[B8] Drake A. Hy J. MacDougal G. Holmes B. Icken L. Schrock J. (2021). Innovations with tele-ultrasound in education sonography: The use of tele-ultrasound to train novice scanners. Ultrasound J. 6, 6. 10.1186/s13089-021-00210-0 PMC7882469 33586112

[B9] Ema R. Wakahara T. Miyamoto N. Kanehisa H. Kawakami Y. (2013). Inhomogeneous architectural changes of the quadriceps femoris induced by resistance training. Eur. J. Appl. Physiol. 113, 2691–2703. 10.1007/s00421-013-2700-1 23949789

[B10] Esformes J. I. Narici M. V. Maganaris C. N. (2002). Measurement of human muscle volume using ultrasonography. Eur. J. Appl. Physiol. 87, 90–92. 10.1007/s00421-002-0592-6 12012082

[B11] Franchi M. V. Fitze D. P. Raiteri B. J. Hahn D. Spörri J. (2020). Ultrasound-derived biceps femoris long head fascicle length: Extrapolation pitfalls. Med. Sci. Sports Exerc. 52, 233–243. 10.1249/MSS.0000000000002123 31403609

[B12] Franchi M. V. Raiteri B. J. Longo S. Sinha S. Narici M. V. Csapo R. (2018). Muscle architecture assessment: Strengths, shortcomings and new Frontiers of *in vivo* imaging techniques. Ultrasound Med. Biol. 44, 2492–2504. 10.1016/j.ultrasmedbio.2018.07.010 30185385

[B13] Franchi M. V. Wilkinson D. J. Quinlan J. I. Mitchell W. K. Lund J. N. Williams J. P. (2015). Early structural remodeling and deuterium oxide-derived protein metabolic responses to eccentric and concentric loading in human skeletal muscle. Physiol. Rep. 3, e12593. 10.14814/phy2.12593 26564061 PMC4673627

[B14] Gans C. Bock W. (1965). The functional significance of muscle architecture--a theoretical analysis. Ergeb. Anat. Entwicklungsgesch. 38, 115–142. 5319094

[B15] Geremia J. M. Baroni B. M. Bini R. R. Lanferdini F. J. de Lima A. R. Herzog W. (2019). Triceps surae muscle architecture adaptations to eccentric training. Front. Physiol. 10, 1456. 10.3389/fphys.2019.01456 31849706 PMC6901927

[B16] Hagoort I. Hortobágyi T. Vuillerme N. Lamoth C. J. C. Murgia A. (2022). Age- and muscle-specific reliability of muscle architecture measurements assessed by two-dimensional panoramic ultrasound. Biomed. Eng. OnLine 21, 15. 10.1186/s12938-021-00967-4 35152889 PMC8842860

[B17] Hopkins W. G. Marshall S. W. Batterham A. M. Hanin J. (2009). Progressive statistics for studies in sports medicine and exercise science. Med. Sci. Sports Exerc. 41, 3–13. 10.1249/MSS.0b013e31818cb278 19092709

[B18] Johnson G. G. R. J. Jelic T. Derksen A. Unger B. Zeiler F. A. Ziesmann M. T. (2022). Accuracy of optic nerve sheath diameter measurements in pocket-sized ultrasound devices in a simulation model. Front. Med. 9, 831778. 10.3389/fmed.2022.831778 PMC8924410 35308521

[B19] Kellis E. Galanis N. Natsis K. Kapetanos G. (2009). Validity of architectural properties of the hamstring muscles: Correlation of ultrasound findings with cadaveric dissection. J. Biomech. 42, 2549–2554. 10.1016/j.jbiomech.2009.07.011 19646698

[B20] Koo T. K. Li M. Y. (2016). A guideline of selecting and reporting intraclass correlation coefficients for reliability research. J. Chiropr. Med. 15, 155–163. 10.1016/j.jcm.2016.02.012 27330520 PMC4913118

[B21] Lieber R. L. Frieden J. (2000). Functional and clinical significance of skeletal muscle architecture. Muscle & Nerve 23, 1647–1666. 10.1002/1097-4598(200011)23:11<1647::aid-mus1>3.0.co;2-m 11054744

[B22] May S. Locke S. Kingsley M. (2021). Reliability of ultrasonographic measurement of muscle architecture of the gastrocnemius medialis and gastrocnemius lateralis. PLOS ONE 16, e0258014. 10.1371/journal.pone.0258014 34587209 PMC8480904

[B23] Miller J. P. Carney M. J. Lim S. Lindsey J. T. (2018). Ultrasound and plastic surgery: Clinical applications of the newest technology. Ann. Plast. Surg. 80, S356–S361. 10.1097/SAP.0000000000001422 29668508

[B24] Narici M. McPhee J. Conte M. Franchi M. V. Mitchell K. Tagliaferri S. (2021). Age-related alterations in muscle architecture are a signature of sarcopenia: The ultrasound sarcopenia index. J. Cachexia Sarcopenia Muscle 12, 973–982. 10.1002/jcsm.12720 34060717 PMC8350200

[B25] Narici M. V. Maganaris C. N. Reeves N. D. Capodaglio P. (2003). Effect of aging on human muscle architecture. J. Appl. Physiol. 95, 2229–2234. 10.1152/japplphysiol.00433.2003 12844499

[B26] Nijholt W. Jager-Wittenaar H. Raj I. S. van der Schans C. P. Hobbelen H. (2020). Reliability and validity of ultrasound to estimate muscles: A comparison between different transducers and parameters. Clin. Nutr. ESPEN 35, 146–152. 10.1016/j.clnesp.2019.10.009 31987109

[B27] Onambele G. L. Narici M. V. Maganaris C. N. (2006). Calf muscle-tendon properties and postural balance in old age. J. Appl. Physiol. 100, 2048–2056. 10.1152/japplphysiol.01442.2005 16455811

[B28] Onambélé G. L. Narici M. V. Rejc E. Maganaris C. N. (2007). Contribution of calf muscle–tendon properties to single-leg stance ability in the absence of visual feedback in relation to ageing. Gait Posture 26, 343–348. 10.1016/j.gaitpost.2006.09.081 17129729

[B29] Oranchuk D. J. Nelson A. R. Storey A. G. Cronin J. B. (2020). Variability of regional quadriceps architecture in trained men assessed by B-mode and extended-field-of-view ultrasonography. Int. J. Sports Physiol. Perform. 15, 430–436. 10.1123/ijspp.2019-0050 31188706

[B30] Paillard T. (2017). Relationship between muscle function, muscle typology and postural performance according to different postural conditions in young and older adults. Front. Physiol. 8, 585. 10.3389/fphys.2017.00585 28861000 PMC5559497

[B31] Puthucheary Z. A. Rawal J. McPhail M. Connolly B. Ratnayake G. Chan P. (2013). Acute skeletal muscle wasting in critical illness. JAMA 310, 1591–1600. 10.1001/jama.2013.278481 24108501

[B32] R Core Team (2020). R: A language and environment for statistical computing. Vienna, Austria: R Foundation for Statistical Computing. Available at: https://www.R-project.org/ .

[B33] Reeves N. D. Maganaris C. N. Longo S. Narici M. V. (2009). Differential adaptations to eccentric *versus* conventional resistance training in older humans: Eccentric resistance training in old age. Exp. Physiol. 94, 825–833. 10.1113/expphysiol.2009.046599 19395657

[B34] Sarto F. Spörri J. Fitze D. P. Quinlan J. I. Narici M. V. Franchi M. V. (2021). Implementing ultrasound imaging for the assessment of muscle and tendon properties in elite sports: Practical aspects, methodological considerations and future directions. Sports Med. 51, 1151–1170. 10.1007/s40279-021-01436-7 33683628 PMC8124062

[B35] Scott J. M. Martin D. S. Ploutz-Snyder R. Matz T. Caine T. Downs M. (2017). Panoramic ultrasound: A novel and valid tool for monitoring change in muscle mass. J. Cachexia Sarcopenia Muscle 8, 475–481. 10.1002/jcsm.12172 28052593 PMC5476852

[B36] Seynnes O. R. Cronin N. J. (2020). Simple Muscle Architecture Analysis (SMA): An ImageJ macro tool to automate measurements in B-mode ultrasound scans. PLOS ONE 15, e0229034. 10.1371/journal.pone.0229034 32049973 PMC7015391

[B37] Stenroth L. Sillanpää E. McPhee J. S. Narici M. V. Gapeyeva H. Pääsuke M. (2015). Plantarflexor muscle–tendon properties are associated with mobility in healthy older adults. J. Gerontol. A Biol. Sci. Med. Sci. 70, 996–1002. 10.1093/gerona/glv011 25733719

[B38] Toscano M. Szlachetka K. Whaley N. Thornburg L. L. (2020). Evaluating sensitivity and specificity of handheld point-of-care ultrasound testing for gynecologic pathology: A pilot study for use in low resource settings. BMC Med. Imaging 20, 121. 10.1186/s12880-020-00518-8 33109134 PMC7590494

[B39] Trezise J. Collier N. Blazevich A. J. (2016). Anatomical and neuromuscular variables strongly predict maximum knee extension torque in healthy men. Eur. J. Appl. Physiol. 116, 1159–1177. 10.1007/s00421-016-3352-8 27076217

[B40] van Alfen N. Gijsbertse K. de Korte C. L. (2018). How useful is muscle ultrasound in the diagnostic workup of neuromuscular diseases? Curr. Opin. Neurol. 31, 568–574. 10.1097/WCO.0000000000000589 30028736

[B41] Weng L. Tirumalai A. P. Lowery C. M. Nock L. F. Gustafson D. E. Von Behren P. L. (1997). US extended-field-of-view imaging technology. Radiology 203, 877–880. 10.1148/radiology.203.3.9169720 9169720

